# Endurance training mitigates obesity-induced hippocampal impairment by enhancing neurotrophin signalling, synaptic plasticity, and cellular responses in a female rat model

**DOI:** 10.1016/j.ibneur.2026.01.006

**Published:** 2026-01-14

**Authors:** Tomáš Kuruc, Karolína Kuchárová, Alexandra Kisucká, Mária Ileninová, Lenka Ihnátová, Katarína Kiss Bimbová, Martina Magurová, Ján Gálik, Nadežda Lukáčová

**Affiliations:** Institute of Neurobiology of Biomedical Research Center, Slovak Academy of Sciences, Šoltésovej 4,6, Košice 040 01, Slovakia

**Keywords:** endurance training, obesity, neurotrophins, hippocampus, signalling pathways

## Abstract

Obesity-related health issues, including cognitive decline linked to hippocampal neurogenesis and neuroplasticity, are gaining more attention as obesity rates rise worldwide. Physical activity is recognized as a potent stimulator of neurotrophic factors. This study examined the impact of six weeks of treadmill training on hippocampal molecular pathways in adult female Zucker diabetic fatty (obese) and Zucker lean rats. Animals were assigned to either treadmill exercise (n = 10) or sedentary control (n = 10) groups. Endurance training (ET) markedly upregulated mRNA expression of brain-derived neurotrophic factor and its receptor. The PI3K/Akt pathway was upregulated only in the trained lean rats and downregulated in the trained obese group compared with sedentary controls. ET elicited divergent effects on neurotrophin-associated PLCγ/PKC/CAMKII signalling between lean and obese groups. Sedentary obese rats primarily utilized the PLCγ/PKC axis, while both trained groups (lean and obese) showed increased CAMKII expression, associated with enhanced synaptic plasticity and memory. Enhanced synaptophysin mRNA indicated improved synaptogenesis and plasticity following ET. Trained obese rats also exhibited reduced expression of the microglial pro-inflammatory marker Iba1, alongside increased markers of oligodendrocyte regeneration and neurofilament expression. Behavioral assessment via the passive avoidance test demonstrated improved learning and memory in trained obese animals. Collectively, these findings suggest that ET may mitigate obesity-induced hippocampal damage, exert neuroprotection, and enhance hippocampal function.

## Highlights

• 6-week endurance training (ET) enhanced hippocampal mRNA levels of BDNF/TrkB.
• ET increased regeneration-related transcripts CNP, PLP1, and NF-l in obese rats.
• ET upregulated CAMKII and synaptophysin mRNAs, supporting plasticity and memory.
• The passive avoidance test showed improvement in learning and memory in obese rats.

## Graphical Abstract

**Figure:**
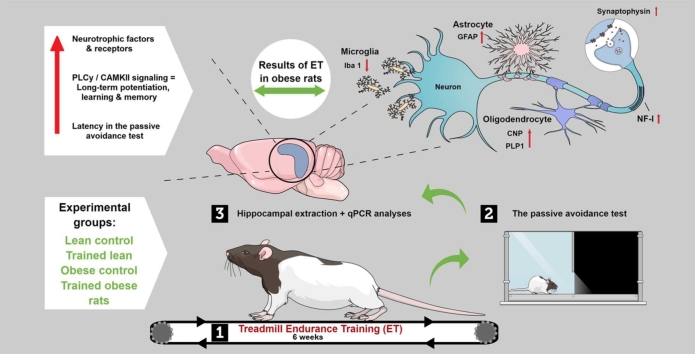
Endurance training is a potent modulator of gene expression changes in the hippocampus. Changes are reflected in the expression profile of neurotrophic factors and their receptors, genes involved in neurotrophic factors signaling, synaptic markers, or regeneration parameters.

## 1 Introduction

Physical inactivity and/or reduced energy expenditure are major public health problems contributing to the development of obesity and associated disorders such as type 2 diabetes, cardiovascular diseases and cancer (Katzmarzyk et al., 2022). Previous reports suggested that physical activity, particularly endurance exercise is a powerful physiological stimulus with a potential to induce widespread structural, neurochemical, mitochondrial, and vascular changes in the brain (Umegaki et al., 2021), which reduce the progression of metabolic problems. Regular endurance exercise benefits the brain by elevating growth factors, particularly the brain-derived neurotrophic factor (BDNF), which crosses the blood-brain barrier in both directions (Pan et al., 1998, Poduslo and Curran, 1996). BDNF provides neuronal support for the brain even during resting conditions following exercise (Hirsch et al., 2018, Wang et al., 2022).

Voluntary exercise and endurance training (ET) have been linked to increased neurogenesis and neuroplasticity in the hippocampus, a region of the brain critical for learning and memory (Karczewski et al., 2022, Lee and Yau, 2020). A mounting body of evidence has demonstrated that ET exerts a stimulatory effect on the proliferation of neural stem cells, the maturation of newly generated neurons, and the promotion of their survival and integration into existing neural circuits (Redila and Christie, 2006, Zhao et al., 2006). However, the level of neuroplasticity in the hippocampus is highly related to the intensity of various training programs (Lin et al., 2012, Lou et al., 2008). Both high- and moderate-intensity ET can enhance hippocampal plasticity by increasing the expression of BDNF and its receptor TrkB (tropomyosin-related kinase B), with subsequent activation of its transcription factor CREB (cAMP response element-binding protein), upregulation of synapsin-I, and other neurotrophic factors like IGF-1 (insulin-like growth factor-1) (Lin et al., 2012, Ploughman et al., 2007). Although an intense training programme can lead to greater increases in BDNF levels than continuous training of moderate intensity (Afzalpour et al., 2015), it could also result in higher levels of corticosterone, which disrupt the process of neurogenesis (Triviño-Paredes et al., 2016). Similarly, the glial cell-derived neurotrophic factor (GDNF) has been reported to increase its level after ET in the brain (Afzalpour et al., 2015). These growth factors mediate their effects by activating TrkB and GDNF family receptor alpha – Gfrα, subsequently triggering specific intracellular signaling cascades. The major signalling pathways ensuring the effect of BDNF and GDNF in different CNS structures are PI3K/Akt (phosphoinositide 3-kinase/protein kinase B), PLCγ/PKC (phospholipase Cγ/protein kinase C), PLCγ/CAMKII (phospholipase Cγ/calcium/calmodulin-dependent kinase II), and Ras/Raf/ERK1/2 (extracellular signal-regulated kinase 1/2) (Sariola and Saarma, 2003, Numakawa et al., 2018, Walker and Xu, 2018, Kiss Bimbova et al., 2022). While neurogenesis and neuroplasticity are interconnected processes, their specific differences in obese versus lean rats may influence metabolic status, which likely contribute to variations in both processes (Liśkiewicz et al. 2020). In fact, it has been reported that obese individuals are more prone to hippocampal neurodegeneration than healthy individuals with subsequent reduction in its volume (Lee and Yau, 2020, Yeshaw et al., 2024).

Unfortunately, the molecular mechanisms underlying these processes, particularly in the hippocampus of obese subjects following exercise, remain largely elusive. Therefore, we aimed to determine the impact of 6-week ET on endogenous stimulation of neurotrophins and activation of signalling pathways in the hippocampus of healthy and obese female rats. Using a treadmill exercise model (Høydal et al., 2007, Samadian et al., 2020), we examined whether training of moderate intensity could influence the gene expression of neurotrophin-associated signalling, specific cell types, and pre- and post-synaptic markers contributing to the modulation of cognitive functions in the hippocampus of obese female subjects.

## 2 Material and methods

### 2.1 Animals and experimental design

Adult female Zucker diabetic fatty (ZDF) rats (290–430 g) and adult Zucker lean (ZL) rats (180–215 g), both aged 3–4 months, were used for this study. The animals were randomly divided into four groups: lean control rats (n = 5), trained lean rats (n = 5), obese control rats (n = 5), and trained obese rats (n = 5). Each experimental group was housed individually in standard cages with lids adapted to hold water bottles and food, allowing for *ad libitum* drinking and eating. Environmental conditions were maintained at a constant temperature of 22°C–24°C, humidity of 45 %–50 %, and a 12-hour light/dark cycle.

The experimental procedures were approved by the State Veterinary and Food Administration of the Slovak Republic (decision No. 4434/16–221/3), as well as by the Animal Use Committee at the Institute of Neurobiology, Biomedical Research Centre of the Slovak Academy of Sciences, in compliance with the EC Council Directive (2010/63/EU) on the use of animals in scientific research. The study adheres to the 2020 AVMA and ARRIVE euthanasia standards (Kilkenny et al., 2010).

### 2.2 Endurance training

Moderate exercise training was scheduled for the trained lean rats and trained obese rats using a rodent treadmill (Treadmill LE 8710; Bioseb, France) with a 0° incline, 5 days a week for 6 weeks (Høydal et al., 2007, Samadian et al., 2020). The intensity and duration of ET varied, beginning in the first week with sessions starting on Monday at 15 min per day at a speed of 9 m/min, continuing through to Friday. Over the following 3 weeks, both speed and duration were gradually increased. In the second week, training was set to 20 min per day at 12 m/min; in the third week, 25 min per day at 15 m/min; and in the fourth week, peak intensity and duration were reached with 30 min per day at 18 m/min. The final 2 weeks maintained this level of intensity and duration (Table 1).

**Table 1:** Training plan for lean and obese rats.

Week of training	Time (min/day)	Speed (m/min)
**1**	15	9
**2**	20	12
**3**	25	15
**4**	30	18
**5**	30	18
**6**	30	18

### 2.3 Behavioral assay - passive avoidance test

**Fig. 1:**
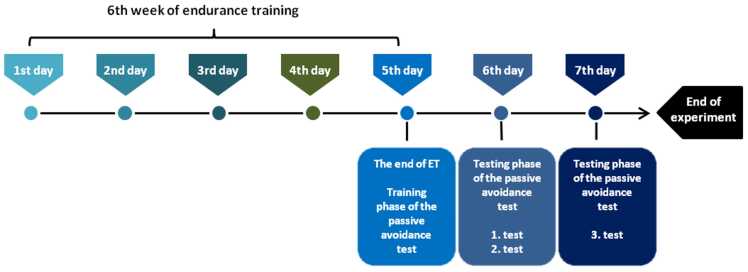
A Schematic representation of the passive avoidance test in relation to the end of ET. The diagram shows the start of the training phase of the passive avoidance test following the final ET session, with testing trials scheduled over the subsequent 2 days. ET – endurance training.

The passive avoidance test is a fear-motivated assessment that evaluates learning and memory in rodents. The experiment was performed at the end of the ET programme (Fig. 1) in accordance with a slightly modified method of Wu et al. (2020), using a passive avoidance box. This apparatus consists of a white (illuminated) compartment and a black (dark) compartment, separated by a guillotine gate (Ugo Basile, Gemonio, Italy). Prior to the experiment, the animals were allowed to habituate in the experimental room for 30 min. They were then placed in the illuminated section to freely explore both areas for 5 min. A small treat was placed in the dark compartment as motivation for the animals to enter that section. After entering the dark compartment during the training phase, the animals received a mild electric shock (0.5 mA), serving as an aversive stimulus associated with darkness. For habituation, the animal was allowed to remain in the dark box for 20 s before being returned to its home cage. Another training trial was performed 4 h later. Following habituation to the illuminated (safe) chamber and the dark (unsafe) compartment, the animals could then decide whether to avoid or re-enter the dark box. The following day, two test trials were conducted, with a final trial performed 24 h later. Animals were placed back in the illuminated section, and the latency time (step-through latency) to enter the dark compartment was recorded for each individual, without administering an electric shock. Test trials ended when the animal entered the dark compartment or remained in the illuminated section for at least 5 min. Data were collected and statistically evaluated. Animals with the longest latencies were considered those that most effectively associated the dark compartment with the aversive stimulus, thus demonstrating faster learning ability.

### 2.4 RT-PCR analysis

Hippocampal tissue samples were thawed for total RNA extraction using Genezol reagent (Thermo Fisher Scientific, MA, USA), following the manufacturer’s instructions. The quality and concentration of isolated RNA were determined by the 260 nm/280 nm ratio using spectrophotometric analysis on the NanoDrop2000c (Thermo Fisher Scientific). For cDNA synthesis, 2000 ng of isolated total RNA was used with the High-Capacity cDNA Reverse Transcription Kit (AB Applied Biosystems, Thermo Fisher Scientific). The process was performed on a T1000™ Thermal Cycler (Bio-Rad, CA, USA), and the CFX96™ Real-Time System (Bio-Rad) was used for cDNA amplification (10 ng per reaction). Primer design was conducted using Geneious software (Biomatters, Ltd., New Zealand) (Table 2). Each primer (1 μL) was used together with Power SYBR Green PCR Master Mix (AB Applied Biosystems, Thermo Fisher Scientific). The amplification protocol was as follows: 10 min at 95°C, followed by 40 cycles alternating denaturation (15 s at 95°C) and annealing/extension (1 min at 60 °C). The relative expression of the analysed targets was determined by normalisation to the reference gene 18S RNA. The calculation of the results was performed by ΔΔCt method.

**Table 2:** Genes used in the experiments along with primer nucleotide sequences.

Target	Forward primer	Reverse primer
**SYP**	ATGGCCACGGACCCAGAGAA	GTTGCCAACCCAGAGCACCA
**LRRC4**	TGTGAAGTGGTTGCTGCCCAA	CCTGCCACGTTGGTCACCAT
**CNPase**	CCTCAGAGCCACCACACATC	CCTTCCTTGGGGCTACAGTG
**NF-I**	AAGGCTAAGACCCTGGAGATCGAAG	GGGATAGTTGGGAATGGGGCTCAA
**PLP1**	TTGGCGACTACAAGACCACC	AATGACACACCCGCTCCAAA
**GAP43**	AGGAAAGGAGAGAAGGCAGG	GCAGGAGAGACAGGGTTCAG
**GFAP**	CAGCTTCGAGCCAAGGAG	TGTCCCTCTCCACCTCCA
**IBA1**	ATCCCAAGTACAGCAGTGATGAGGA	AAATAGCTTTCTTGGCTGGGGGAC
**BDNF**	TGCAGGGGCATAGACAAAAGG	CTTATGAATCGCCAGCCAATTCTC
**GDNF**	GCCACCATCAAAAGACTGAAAAGG	AGAGAGAGGAACCGGCAAGC
**Gfrα**	CTGGATTTGCTGATGTCCGC	CTTTCTTCATGCCCCGCTTG
**TRKB**	GCATTTTGCACCAACCATCAC	CACAGTGAATGGGATGCACC
**RBFox3**	CTTCCAGGGTCGTGTATCAG	CTCTACCATAACTGTCACTGTAAG
**PI3K**	AACACAGAAGACCAATACTC	TTCGCCATCTACCACTAC
**Akt**	GTGGCAAGATGTGTATGAG	CTGGCTGAGTAGGAGAAC
**RAF**	CGTTCAGCTTCCAGTCCGAT	CTTCACACAGTCAGCCACCA
**ERK1**	TCCGGGGCCTCAAGTACATA	AAGCATGATCTCTGGGGCTC
**ERK2**	AAGCCTTCCAACCTCCTGC	ATGCAGCCCACAGACCAAT
**PLCγ**	ATAAGAAGCTGGCTGAGGGC	ATTTCCCTGGTCACTGCTGG
**PKC**	GATGAAATGCGACACCTGCG	CGTAAGGATCCGAAAGCCCA
**CAMKII**	TACACGAAGATGTGCGACCC	GTGATGCGGATGTAGGCGAT
**18sRNA**	GACCATAAACGATGCCGACT	GTGAGGTTTCCCGTGTTGAG

### 2.5 Tissue collection

At the end of the experiment, the animals were euthanised under isoflurane anaesthesia. The hippocampus was extracted, frozen in liquid nitrogen, and stored at −80°C for further analyses.

### 2.6 Statistical analysis

Statistical analyses of all obtained data were performed using GraphPad Prism software version 8.01 (GraphPad Software, La Jolla, CA, USA). Prior to analysis of variance (ANOVA), normality was assessed using the Kolmogorov–Smirnov test and the Shapiro–Wilk test. All data sets were tested for normality (p > 0.05), allowing analysis of RT-PCR data using one-way ANOVA followed by Tukey’s post hoc test. This involved comparing individual data sets plotted as fold changes relative to the lean control, which served as the baseline. Two-way ANOVA with Dunnett’s multiple comparisons test was used to analyse data on body weight changes and behavioural assays during the experiment. Correlation analyses were performed using Pearson correlation test. Differences were considered significant when the p-value was < 0.05.

## 3 Results

### 3.1 Impact of ET on body weight

**Fig. 2:**
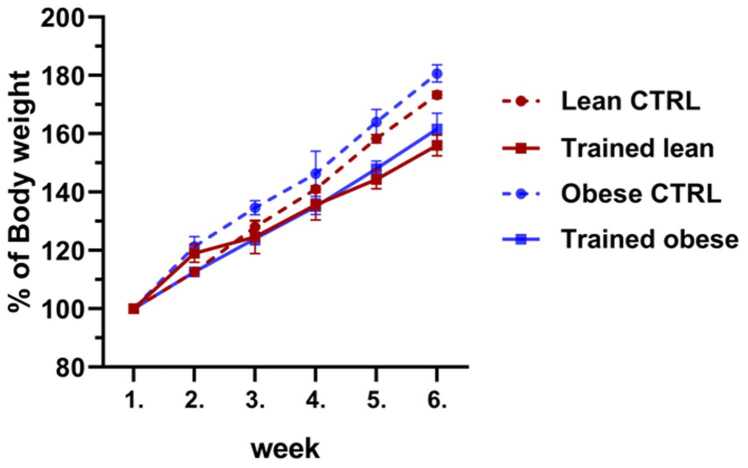
Percentage changes in body weight of individual experimental groups during 6 weeks of ET on a treadmill. Results were collected after 6 weeks of ET and evaluated using two-way ANOVA with Tukey’s post hoc test. Results are presented as mean ± SEM (n = 5).

Because exercise increases energy expenditure, we monitored changes in body weight between the trained and sedentary groups during the training programme. As expected, the greatest percentage of body weight gain was observed in the obese control group (Fig. 2). However, no significant differences were detected between the trained and sedentary groups, although there was a general tendency toward weight reduction following ET.

### 3.2 Passive avoidance test

**Fig. 3:**
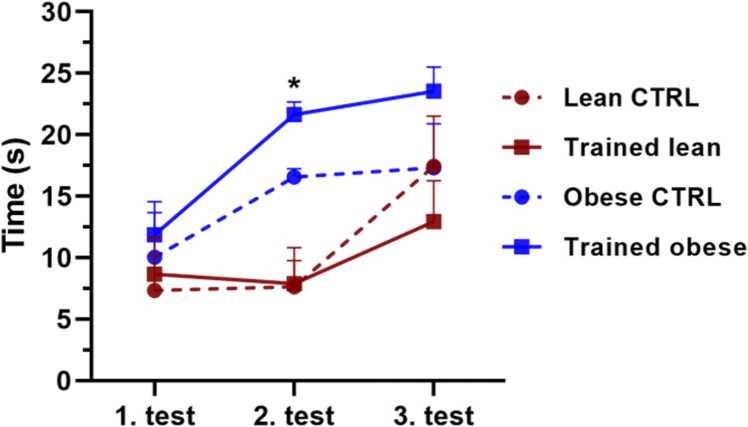
Passive avoidance test to assess learning and memory. The figure shows changes in latency among the experimental groups: lean CTRL, lean trained, obese CTRL, and obese trained animals. Results were collected after 6 weeks of ET and evaluated using two-way ANOVA with Tukey’s post hoc test. Results are presented as mean ± SEM (n = 5). Statistical significance is indicated as follows: *p < 0.05 vs lean control.

The passive avoidance test is a well-established behavioural assay used to assess long-term memory. The results presented in Fig. 3 show differences in latency among the experimental groups across three individual tests. Only the second test revealed significant differences between groups. A significant increase in latency (p = 0.0318) was observed in trained obese animals compared with lean controls. Although the recorded latencies were lower than expected and intergroup differences were not highly pronounced, the trained obese group demonstrated the most notable improvements in latency during both the second and third tests.

### 3.3 Impact of endurance training on gene expression of BDNF, GDNF, TrkB and Gfrα in the hippocampus of lean and obese rats

**Fig. 4:**
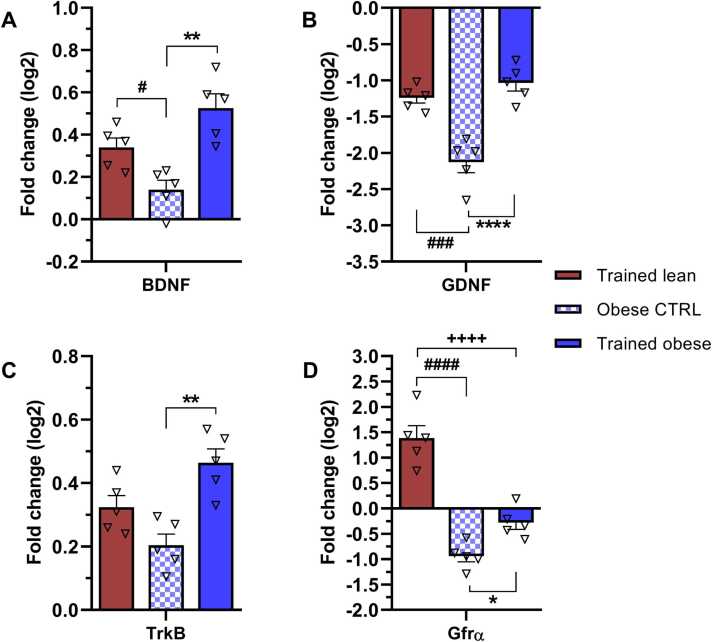
Changes in hippocampal gene expression of the neurotrophic factors (A) BDNF, (B) GDNF, and their corresponding receptors (C) TrkB and (D) Gfrα in response to endurance training and obesity. mRNA levels are expressed as ∆∆Ct values relative to the lean control, which was set as the baseline. Bars represent fold change (log2) relative to the lean control. Data were collected after 6 weeks of treadmill endurance training and statistically evaluated using one-way ANOVA followed by Tukey’s post hoc test. Results are presented as mean ± SEM (n = 5). Statistical significance is indicated as follows: *p < 0.05, **p < 0.01, and ***p < 0.001 for obese CTRL vs trained obese; + ++ +p < 0.0001 for trained lean vs trained obese; and #p < 0.05 and ###p < 0.001 for trained lean vs obese CTRL. BDNF – brain-derived neurotrophic factor; GDNF – glial cell-derived neurotrophic factor; TrkB – tropomyosin-related kinase B; Gfrα – GDNF family receptor alpha.

We first examined differences in hippocampal mRNA levels of BDNF, GDNF, and their receptors (TrkB and Gfrα) across all experimental groups, using the lean control group as the baseline (Fig. 4). In general, hippocampal gene expression of BDNF and the TrkB receptor was increased (by 0.1- to 0.5-fold change). The greatest increases in expression of these genes were observed in the trained obese group (0.5-fold change, respectively) (Fig. 4A, C). By contrast, GDNF (Fig. 4B) and its receptor Gfrα (Fig. 4D) were markedly downregulated in all experimental groups compared with the lean control, except for the Gfrα mRNA level in the trained lean group (1.3-fold change). The most prominent decreases in GDNF (−2.1-fold change) and Gfrα (−1.0-fold change) expression were observed in the obese control group (Fig. 4B, D).

Compared with obese controls, we noted a significant increase in BDNF mRNA levels in both trained groups (lean, p = 0.0496; obese, p = 0.0016) (Fig. 4A). Although gene expression analysis for the TrkB receptor (Fig. 4C) revealed trends similar to those seen for its ligand BDNF across all groups, only the trained obese group showed a significant increase in TrkB mRNA levels (p = 0.0012). Furthermore, 6 weeks of ET resulted in a significant increase in GDNF mRNA levels in the trained lean group (p = 0.0004) and a significant recovery in the trained obese group (p < 0.0001) (Fig. 4B), although these levels remained below baseline. A comparable pattern was observed in the expression of Gfrα in both trained groups (p = 0.0489; p < 0.0001 in lean) and in obese animals (Fig. 4D). These results suggest that obese rats, which possess a mutated leptin receptor (Phillips et al., 1996), exhibit downregulation of hippocampal GDNF and Gfrα expression compared with lean rats. Additionally, 6 weeks of ET effectively reversed this decrease in expression. Therefore, regular exercise may positively impact gene expression, potentially upregulating or partially restoring the previously reduced expression of neurotrophic factors and their receptors.

### 3.4 Alterations in gene expression of components within neurotrophin-related signalling pathways

**Fig. 5:**
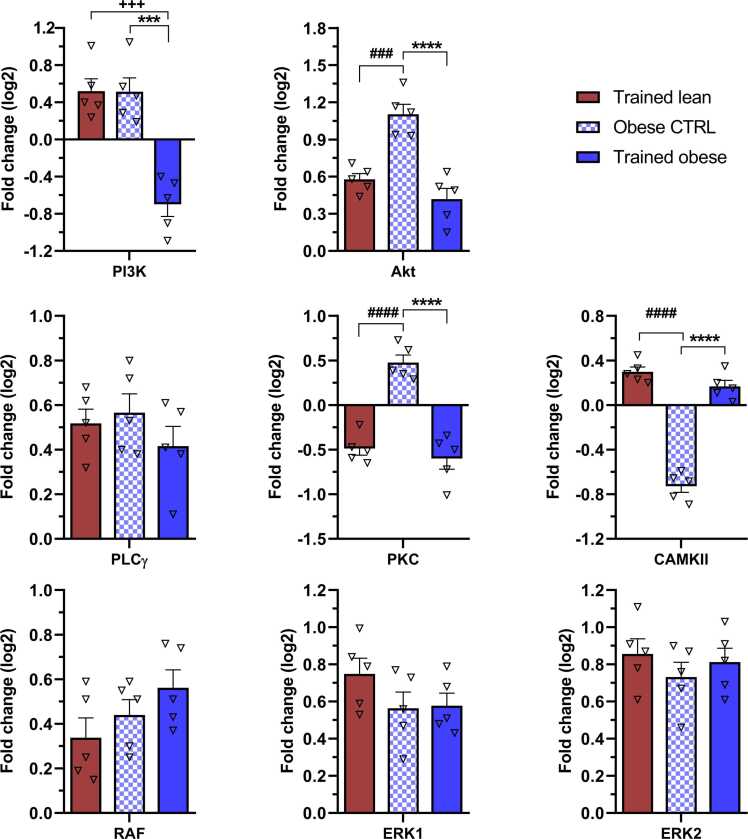
Effect of endurance training and obesity on the modulation of gene expression in the (A) PI3K/ (B) Akt, (C) PLCγ/ (D) PKC/ (E) CAMKII, and (F) RAF/ (G) ERK1/ (H) ERK2 signalling pathways. mRNA levels are expressed as ∆∆Ct values relative to the lean control, which was set as the baseline. Bars in the graphs represent fold change (log2) relative to the lean control. Data were collected after 6 weeks of treadmill endurance training and statistically evaluated using one-way ANOVA followed by Tukey’s post hoc test. Results are presented as mean ± SEM (n = 5). Statistical significance is indicated as follows: ***p < 0.001 and ****p < 0.0001 for obese CTRL vs trained obese; + ++p < 0.001 for trained lean vs trained obese; and ###p < 0.001 and ####p < 0.0001 for trained lean vs obese CTRL. PI3K – phosphoinositide 3-kinase; Akt – protein kinase B; PLCγ – phospholipase Cγ; PKC – protein kinase C; CAMKII – calcium/calmodulin-dependent kinase II; RAF – proto-oncogene serine/threonine-protein kinase; ERK1/2 – extracellular signal-regulated kinase ½.

Both BDNF and GDNF mediate their effects by activating specific intracellular signalling pathways responsible for various functions within neuronal tissues. Therefore, we further evaluated whether endogenous stimulation of these neurotrophins by 6 weeks of ET contributes to the upregulation of mRNAs in the PI3K/Akt signalling pathway, associated with cell survival. As shown in Fig. 5A, the trained lean and obese control groups exhibited approximately 0.5-fold upregulation of PI3K mRNA levels compared with the lean control, whereas the trained obese group demonstrated suppression of PI3K expression, with a fold change of approximately −0.6. Statistical comparisons revealed a significant reduction in PI3K expression in the trained obese group compared with both the trained lean (p = 0.0001) and obese control (p = 0.0001) groups. Relative to the lean control, Akt—a key downstream target of PI3K—was upregulated (approximately 0.5-fold change) at the mRNA level in both trained groups (Fig. 5B). The obese control group displayed a more robust increase in relative mRNA levels, exceeding a 1.0-fold change. This increase in the obese control group was significantly greater than in the trained lean (p = 0.0008) and trained obese (p < 0.0001) groups. Notably, trained obese rats showed significant downregulation of mRNA levels for both PI3K and Akt compared to the obese control group (Fig. 5A and B). These data suggest that ET can promote gene expression of components within the PI3K/Akt signalling pathway in the hippocampus of lean rats, but not as effectively in obese animals.

It is widely known that activation of the PLCγ/PKC and PLCγ/CAMKII signalling pathways in the hippocampus is crucial for neuroplasticity, learning, and memory. PLCγ gene expression, the first downstream target following TrkB and/or Gfrα activation, was elevated in all experimental groups, exceeding a 0.4-fold change compared with the lean control group (Fig. 5C). Although PKC is a critical mediator of neuronal plasticity (Fink, 2002), it may also regulate microglial polarisation during certain pro-inflammatory processes (Kiss Bimbova et al., 2022). As shown in Fig. 5D, the increase in PKC mRNA levels occurred exclusively in the obese control group (0.5-fold change) relative to the lean control. By contrast, animals that underwent treadmill training exhibited comparable but negative fold-change values (−0.5), with statistically significant differences (p < 0.0001) observed in both trained groups compared with obese controls. These findings indicate that obesity upregulates PLCγ/PKC signalling in the hippocampus and that ET contributes to regulation of this pathway in both lean and obese animals. Conversely, opposite trends were observed in CAMKII gene expression. The PLCγ/CAMKII signalling pathway is known to play a key role in maintaining dendritic spine architecture and synaptic plasticity in the hippocampus (Yasuda et al., 2022, Curtis et al., 2023). The obese control group exhibited a marked decrease in CAMKII mRNA levels (−0.7-fold change) compared with the lean control (Fig. 5E). However, ET positively modulated CAMKII gene expression in both the lean (0.3-fold change) and trained obese (0.2-fold change) groups. Statistical analyses revealed that CAMKII, which is closely associated with Ca^2+^ signalling, was significantly upregulated (p < 0.0001) after 6 weeks of ET in both lean and obese groups. These results suggest that Ca^2+^ signalling may play a primary role in the hippocampus of trained animals.

The RAF/ERK1/2 signalling pathway is well known for its involvement in processes such as proliferation, differentiation, and pro-survival activity. Conversely, this signalling pathway also plays a key pro-apoptotic role in the development of various pathological conditions (Feng et al., 2016, Ohkura et al., 2019). As shown in Fig. 5F–H, endurance training led to a progressive increase in mRNA levels of RAF (0.35-fold change), ERK1 (0.75-fold change), and ERK2 (0.85-fold change) in the trained lean group compared with the lean control. Although this trend was not as pronounced in the trained obese group, it also exhibited consistent upregulation across all genes, with no values dropping below a 0.5-fold change. Interestingly, even the obese control group showed more than a 0.4-fold increase in mRNA levels of each gene relative to the lean control. However, no significant intergroup differences were observed. Based on these findings, we can infer that each component of the RAF/ERK1/2 signalling pathway is susceptible to modulation not only by ET but also by obesity itself, when compared with lean controls.

### 3.5 Correlation analyses based on relative gene expression of neurotrophic factors, their receptors and intracellular signalling components

Correlation analyses revealed group-specific patterns. ET induced high positive correlations (++++) between BDNF expression and all components of the RAF/ERK1/ERK2 signalling axis, particularly in trained obese subjects. Additionally, the trained obese group demonstrated negligible (+), moderate (+++) and high positive (++++) correlations of BDNF with components of the PLCγ/PKC and PLCγ/CAMKII pathways. In contrast, these correlations were predominantly negative in the obese control group, except for ERK2 (+++++) and PKC (+++) (Table 3). A divergent pattern emerged for GDNF, where the obese control group showed high to very high positive correlations with RAF (++++) and ERK1 (+++++). Nevertheless, the trained obese group maintained moderate to high positive correlations with RAF (+++) and ERK2 (++++). Receptor expression patterns diverged from their respective neurotrophins; no comparable correlation trends were observed. The most significant difference was observed in the trained obese group, where the correlations of the TrkB receptor and RAF/ERK1/ERK2 showed negative values, in contrast to those of BDNF. The only consistent highly positive receptor-pathway correlations occurred between TrkB and the PI3K/AKT axis in the obese control group.

**Table 3:** Correlation analyses of neurotrophic factors and receptors versus intracellular signalling pathway components across experimental groups.

		PI3K	AKT	PLCγ	PKC	CAMKII	RAF	ERK1	ERK2
**BDNF**	Trained lean	+ +	-	+	+ +	-	+	+ ++ +	-
	Obese CTRL	+ +	+	- - -	+ ++	-	- - -	- -	+ ++ ++*
	Trained obese	-	+ +	+	+ ++	+ ++ +	+ ++ +	+ ++ +	+ ++ +
**GDNF**	Trained lean	-	-	+	+	+	+	+ +	- - - -
	Obese CTRL	+	+ ++	+ ++ +	+	- - -	+ ++ +	+ ++ ++*	- -
	Trained obese	- - -	- - -	+ +	+ ++	+ +	+ ++	+ +	+ ++ +
**TrkB**	Trained lean	+ ++	+	+	+ +	-	+ +	+ ++	+
	Obese CTRL	+ ++ +	+ ++ +	-	+ ++ ++*	-	- -	+	+ ++
	Trained obese	+ ++ ++ ***	-	- -	-	+ +	-	- -	- -
**Gfrα**	Trained lean	- -	- -	+	-	-	- -	+ +	- - - - -*
	Obese CTRL	+	+ +	-	+ +	- - - -	+	+	+ ++ +
	Trained obese	+	-	- - - -	+ ++ +	+	-	- -	-

The data were calculated using regression analysis of relative gene expressions. According to Mukaka (2012), Pearson’s correlation coefficients (r) were divided into five categories based on the size of the correlation and interpreted as follows: 0.9–1.0 (-0.9 to −1.0) - Very high positive (negative) correlation (+++++/- - - - -); 0.7–0.9 (-0.7 to −0.9) - High positive (negative) correlation (++++/- - - -), 0.5–0.7 (-0.5 to −0.7) - Moderate positive (negative) correlation (+++/- - -), 0.3–0.5 (-0.3 to −0.5) - Low positive (negative) correlation (++/- -), and 0.0–0.3 (0.0 to −0.3) - Negligible positive (negative) correlation (+/-). Statistical significance is indicated as follows: *p < 0.05 and ***p < 0.001

These findings suggest that ET preferentially enhances positive associations between neurotrophic factors BDNF, GDNF, and the RAF/ERK1/ERK2 and PLCγ/PKC/CAMKII signalling pathways, particularly in obese trained subjects. Conversely, positive relationships involving the PI3K/AKT axis, neurotrophins, and their receptors were demonstrated predominantly in the obese control group.

### 3.6 Different effects of ET on synaptic markers and post-mitotic neurons in lean and obese animals

**Fig. 6:**
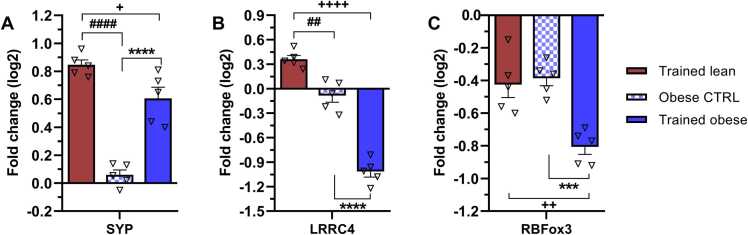
Modulation of mRNAs specific to (A) presynaptic (SYP), (B) postsynaptic (LRRC4), and (C) post-mitotic neuronal (RBFox3) markers as a result of endurance training and obesity. mRNA levels are expressed as ∆∆Ct values relative to the lean control, which was set as the baseline. Bars in the graphs represent fold change (log2) relative to the lean control. Data were collected after 6 weeks of treadmill endurance training and statistically evaluated using one-way ANOVA followed by Tukey’s post hoc test. Results are presented as mean ± SEM (n = 5). Statistical significance is indicated as follows: ***p < 0.001 and ****p < 0.0001 for obese CTRL vs trained obese; +p < 0.05, + +p < 0.01, and + ++ +p < 0.0001 for trained lean vs trained obese; and ##p < 0.01 and ####p < 0.0001 for trained lean vs obese CTRL. SYP – synaptophysin; LRRC4 – leucine-rich repeat-containing 4; RBFox3 – RNA binding fox-1 homolog 3.

Modulation of mRNA levels of presynaptic (synaptophysin -SYP) and postsynaptic (LRRC4) markers may reflect changes associated with synaptogenesis, including neurite sprouting, increased connectivity, or synaptic plasticity. As shown in Fig. 6A and B, the trained lean group was the only group capable of positively modulating the gene expression of SYP (0.9-fold change) and LRRC4 (0.3-fold change) compared with the lean control (baseline). By contrast, trained obese animals effectively upregulated only SYP mRNA (0.6-fold change), while LRRC4 expression remained unchanged. The obese control group maintained both transcript levels at values comparable to the lean control (Fig. 6A and B). Consequently, we observed significant differences in the expression profiles of SYP (p < 0.0001) and LRRC4 (p < 0.0001) between obese groups. Overall, ET significantly favoured lean animals in upregulating both synaptic markers—SYP (p = 0.0228) and LRRC4 (p < 0.0001)—when compared with their obese counterparts.

Fig. 6C shows a decrease in RBFox3 gene expression, a marker of post-mitotic neurons, across all experimental groups relative to the lean control. The most pronounced suppression (−0.8-fold change) occurred in the trained obese group, resulting in significant differences when compared with the trained lean (p = 0.0018) and obese control (p = 0.0008) groups.

These findings suggest that ET does not enhance gene expression of post-mitotic neurons, as indicated by RBFox3 levels, but does modulate synaptic plasticity. This dichotomy highlights the complex effects of ET on neural health, potentially indicating that while ET may not promote the generation or maintenance of post-mitotic neurons, it can still enhance synaptic function and connectivity.

### 3.7 Effect of ET on changes in mRNA levels of mature and immature oligodendrocyte markers, together with regeneration parameters

Oligodendrocytes represent a crucial component of the neuronal support system, particularly in association with axon myelination. Distinct stages of oligodendrocyte maturation are characterised by the differential expression of specific molecular markers prevalent at each stage. Based on this, we investigated changes in the gene expression of CNP and PLP1 (immature/mature oligodendrocytes) alongside NF-l and GAP43 (regeneration parameters).

**Fig. 7:**
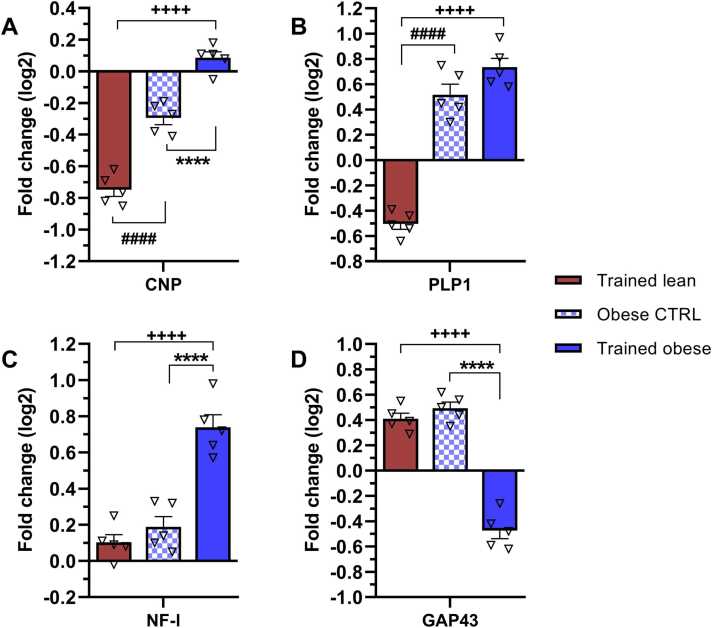
Modulation of mRNA levels of (A) CNP (immature oligodendrocytes) and (B) PLP1 (mature oligodendrocytes), along with (C) NF-l and (D) GAP43 mRNAs representing regeneration parameters, as a result of endurance training and obesity. mRNA levels are expressed as ∆∆Ct values relative to the lean control, which was set as the baseline. Bars in the graphs represent fold change (log2) relative to the lean control. The presented data were collected after 6 weeks of treadmill endurance training and statistically evaluated using one-way ANOVA followed by Tukey’s post hoc test. Results are presented as mean ± SEM (n = 5). Statistical significance is indicated as follows: ****p < 0.0001 for obese CTRL vs trained obese, + ++ +p < 0.0001 for trained lean vs trained obese, ####p < 0.0001 for trained lean vs obese CTRL. CNP – 2′,3′-cyclic-nucleotide 3′-phosphodiesterase; PLP1 – proteolipid protein 1; NF-l – neurofilament light chain; GAP43 – growth-associated protein 43.

The results shown in Fig. 7A and B indicate that lean and obese trained animals exhibited opposite transcript profiles for both oligodendrocyte markers compared with the lean control group. While ET resulted in a notable decline in CNP (−0.7-fold change) and PLP1 (−0.5-fold change) mRNA levels in the lean group, obese animals exhibited increased expression of both genes above baseline, with the highest increase observed in PLP1 (0.7-fold change). Additionally, the obese control group showed upregulation of PLP1 (0.5-fold change), whereas CNP was downregulated (−0.3-fold change). These differences in CNP and PLP1 gene expression yielded statistically significant differences (p < 0.0001) between the trained groups. In obese animals, ET tended to increase PLP1 gene expression; however, it significantly restored only CNP mRNA levels. Thus, oligodendrocyte markers appear more likely to be expressed in obese animals following ET than in lean ones.

Gene expression levels of regeneration parameters, represented by NF-l and GAP43, are shown in Fig. 7C and D. Significant modulation of NF-l and GAP43 gene expression was observed exclusively in the trained obese group when compared with their sedentary controls (p < 0.0001) as well as trained lean animals (p < 0.0001). Interestingly, ET in obese animals affected GAP43 gene expression in an opposite manner to that of NF-l.

In conclusion, ET appears to be more beneficial for obese animals than for their lean counterparts in terms of enhancing expression of both immature and mature oligodendrocyte markers, as well as the regeneration marker NF-l.

### 3.8 Changes in astrocyte- and microglia-specific mRNAs induced by ET

**Fig. 8:**
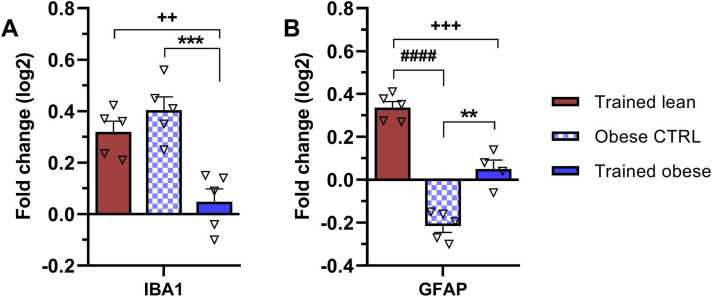
Modulation of mRNA levels of (A) IBA1 (microglia) and (B) GFAP (astrocytes) as a result of endurance training and obesity. mRNA levels are expressed as ∆∆Ct values relative to the lean control which was set as the baseline. Bars in the graphs represent fold change (log2) relative to the lean control. Data were collected after 6 weeks of treadmill endurance training and statistically evaluated using one-way ANOVA followed by Tukey’s post hoc test. Results are presented as mean ± SEM (n = 5). Statistical significance is indicated as follows: **p < 0.01 and ***p < 0.001 for obese CTRL vs trained obese, + +p < 0.01 and + ++p < 0.001 for trained lean vs trained obese, and ####p < 0.0001 for trained lean vs obese CTRL. IBA1 – ionised calcium-binding adaptor molecule 1; GFAP – glial fibrillary acidic protein.

Both astrocytes and microglial cells play important roles in synaptic plasticity. While astrocytes contribute to maintenance and metabolic support, microglia are responsible for pruning damaged and unnecessary synapses (Hong et al., 2016). In the present study, RT-PCR analysis revealed increased expression of both IBA1 (0.3-fold change) and GFAP mRNAs (0.35-fold change) 6 weeks after ET in lean animals compared with their sedentary controls (Fig. 8A and B). This modulation was significantly greater than that observed in the trained obese group for both IBA1 (p = 0.0045) and GFAP (p = 0.0005).

Furthermore, obese animals showed significant suppression of IBA1 mRNA levels (p = 0.0005) in response to ET, along with a substantial increase in GFAP gene expression (p = 0.0019) compared with the obese control group. Obesity alone had the strongest effect on IBA1 upregulation (0.4-fold change) and a reduction of GFAP transcript levels below baseline. These findings suggest a potential neuroprotective effect of ET in obese animals: suppression of microglial activation may reflect reduced pathological conditions, while increased astrocyte gene expression could provide enhanced neuronal support. The resulting gene-related effects are summarised in Table 4.

**Table 4:** Summary of statistically significant changes in expression profiles of individual genes.

Groups		Trained lean vs Trained obese	Trained lean vs Obese CTRL	Trained obese vs Obese CTRL
Genes				
**Neurotrophins and receptors**	BDNF	**−−**	**↑**	**↑**
	TrkB	**−−**	**−−**	**↑**
	GDNF	**−−**	**↑**	**↑**
	GFrα	**↑**	**↑**	**↑**
**Signalling pathway components**	PI3K	**↑**	**−−**	**↓**
	Akt	**−−**	**↓**	**↓**
	PLCγ	**−−**	**−−**	**−−**
	PKC	**−−**	**↑**	**↑**
	CAMKII	**−−**	**↑**	**↑**
	RAF	**−−**	**−−**	**−−**
	ERK1	**−−**	**−−**	**−−**
	ERK2	**−−**	**−−**	**−−**
**Synaptic markers**	SYP	**↑**	**↑**	**↑**
	LRRC4	**↑**	**↑**	**↓**
**Regeneration markers**	CNP	**↓**	**↓**	**↑**
	PLP1	**↓**	**↓**	**−−**
	NF-l	**↓**	**−−**	**↑**
	GAP43	**↑**	**−−**	**↓**
**Cellular components**	IBA1	**↑**	**−−**	**↓**
	GFAP	**↑**	**↑**	**↑**
	RBFox3	**↑**	**−−**	**↓**

### 3.9 Correlations between the gene expression of signalling molecules and synaptic, regeneration and neural markers

The PI3K/AKT signalling pathway exhibited group-specific correlation patterns with genes involved in myelination (CNP and PLP1). These findings were driven mainly by the PI3K component within both trained groups, where correlation magnitudes ranged from low to high positive values. Similarly, AKT demonstrated positive correlations with CNP (++) and PLP1 (++++), but this was restricted only to the trained lean group. Surprisingly, the obese controls showed high to very high positive correlations between PI3K/AKT and CNP. PI3K/AKT also showed a consistent moderate positive correlations (+++) with the microglial activation marker IBA1 in trained lean and AKT across all experimental groups (Table 5).

**Table 5:** Correlation analyses of intracelullar PI3K/AKT signalling pathway components versus synaptic, regeneration, and neural markers across experimental groups.

		SYP	LRRC4	RBFox3	CNP	PLP1	NF-l	GAP43	IBA1	GFAP
**PI3K**	Trained lean	+	-	-	+ ++	+ +	-	-	+ ++	+ ++
	Obese CTRL	+	+ ++	-	+ ++ +	-	+	+ ++	- -	+
	Trained obese	+ +	-	- - -	+ ++	+ ++ +	- - -	- - - -	+	- - - -
**AKT**	Trained lean	+	+	+ ++ ++*	+ +	+ ++ +	-	+	+ ++	- - -
	Obese CTRL	+ ++ +	-	+ ++	+ ++ ++*	+	+ +	+ ++ +	+ ++	+
	Trained obese	- - -	+	-	-	- -	+	+	+ ++	+ +

The data were calculated using regression analysis of relative gene expressions. According to Mukaka (2012), Pearson’s correlation coefficients (r) were divided into five categories based on the size of the correlation and interpreted as follows: 0.9–1.0 (-0.9 to −1.0) - Very high positive (negative) correlation (+++++/- - - - -); 0.7–0.9 (-0.7 to −0.9) - High positive (negative) correlation (++++/- - - -), 0.5–0.7 (-0.5 to −0.7) - Moderate positive (negative) correlation (+++/- - -), 0.3–0.5 (-0.3 to −0.5) - Low positive (negative) correlation (++/- -), and 0.0–0.3 (0.0 to −0.3) - Negligible positive (negative) correlation (+/-). Statistical significance is indicated as follows: *p < 0.05.

A highly positive correlation (++++) was identified also between the PLP1 and CAMKII, specifically within both trained groups, uderscoring a group-specific pattern. In contrast, the obese control group exhibited a pronounced negative correlation (----) between the expression of these genes. Together with findings involving the PI3K/AKT pathway, the CAMKII, branch of the PLCγ/PKC/CAMKII signalling highlights the significance of ET in modulating myelination processes *via* these pathways. Furthermore, the involvement of ET within the PLCγ/PKC/CAMKII signalling pathway also showed group-dependent correlations with the postsynaptic marker LRRC4, which was again group-dependent. Notably, a very high positive correlation (++++) was observed between LRRC4 and PKC in the obese group, whereas CAMKII exhibited a moderate correlation with LRRC4 in the trained lean group (Table 5, Table 6).

**Table 6:** Correlation analyses of intracelullar PLCγ/PKC/CAMKII signalling pathway components versus synaptic, regeneration, and neural markers across experimental groups.

		SYP	LRRC4	RBFox3	CNP	PLP1	NF-l	GAP43	IBA1	GFAP
**PLCγ**	Trained lean	- - -	+ +	-	-	+	- - -	-	+	+ +
	Obese CTRL	+ ++	- - - -	+ ++ ++*	+ +	+	-	+	+ ++ +	-
	Trained obese	+	- - -	-	- - - -	-	+ ++ +	+ ++	+	+ ++ ++
**PKC**	Trained lean	-	+	+	+ +	+ ++	- -	+	+ ++	+
	Obese CTRL	+	+ ++	-	+ ++ +	+	+	+ ++ +	-	+ +
	Trained obese	- - -	+ ++ ++*	+ ++ +	+ +	+	+	+ +	+ +	-
**CAMKII**	Trained lean	-	+ ++	+ ++ ++*	+	+ ++ +	- - -	-	+ ++	- -
	Obese CTRL	-	+	- -	-	- - - -*	-	-	- - - -	- - -
	Trained obese	+	+	-	+	+ ++ +	+ +	+ +	+ ++	+ +
	Trained obese	-	+	+	- -	+	+ ++ ++*	+ ++ ++*	+ +	+ ++ +

The data were calculated using regression analysis of relative gene expressions. According to Mukaka (2012), Pearson’s correlation coefficients (r) were divided into five categories based on the size of the correlation and interpreted as follows: 0.9–1.0 (-0.9 to −1.0) - Very high positive (negative) correlation (+++++/- - - - -); 0.7–0.9 (-0.7 to −0.9) - High positive (negative) correlation (++++/- - - -), 0.5–0.7 (-0.5 to −0.7) - Moderate positive (negative) correlation (+++/- - -), 0.3–0.5 (-0.3 to −0.5) - Low positive (negative) correlation (++/- -), and 0.0–0.3 (0.0 to −0.3) - Negligible positive (negative) correlation (+/-). Statistical significance is indicated as follows: *p < 0.05.

RAF/ERK1/ERK2 signalling also demonstrated significant correlations associated with the expression of the regenerative markers NF-l and GAP43, particularly in trained obese animals. Each component of this pathway exhibited high (++++) to very high positive (+++++) correlations with the relative expression of both genes. Concurrently, a similarly significant association was observed with GFAP, a marker of activated astrocytes, suggesting a coordinated role of this signalling cascade in neuronal regeneration and astroglial activation (Table 7).

**Table 7:** Correlation analyses of extracelullar RAF/ERK1/ERK2 signalling pathway components versus synaptic, regeneration, and neural markers across experimental groups.

		SYP	LRRC4	RBFox3	CNP	PLP1	NF-l	GAP43	IBA1	GFAP
**RAF**	Trained lean	-	+ +	+ ++	+ +	+ ++ +*	- -	-	+ ++ +	-
	Obese CTRL	+	- - - -*	+ ++ +*	+	+ +	- -	-	+ ++	+
	Trained obese	-	+	-	- -	+ +	+ ++ +	+ ++ +	+ +	+ ++ +
**ERK1**	Trained lean	+ ++	- - - -	- - -	+ ++	+	+ +	+ ++ +	-	-
	Obese CTRL	+ ++	- - - -	+ ++ ++**	+ ++	+	-	+	+ ++ +	+
	Trained obese	-	-	-	- - -	+	+ ++ ++**	+ ++ +	+ +	+ ++ ++
**ERK2**	Trained lean	+ ++	-	+ ++	+ +	+ +	+ ++	- -	+ ++ +	+
	Obese CTRL	-	+ ++ +	- - -	+	+ ++	+ ++ +	+ ++	+	+ +
	Trained obese	-	+	+	- -	+	+ ++ ++*	+ ++ ++*	+ +	+ ++ +

The data were calculated using regression analysis of relative gene expressions. According to Mukaka (2012), Pearson’s correlation coefficients (r) were divided into five categories based on the size of the correlation and interpreted as follows: 0.9–1.0 (-0.9 to −1.0) - Very high positive (negative) correlation (+++++/- - - - -); 0.7–0.9 (-0.7 to −0.9) - High positive (negative) correlation (++++/- - - -), 0.5–0.7 (-0.5 to −0.7) - Moderate positive (negative) correlation (+++/- - -), 0.3–0.5 (-0.3 to −0.5) - Low positive (negative) correlation (++/- -), and 0.0–0.3 (0.0 to −0.3) - Negligible positive (negative) correlation (+/-). Statistical significance is indicated as follows: *p < 0.05; **p < 0.01.

## 4 Discussion

Aerobic ET provides numerous health benefits, including favourable effects on body weight, glucose regulation, blood pressure control, and overall improvements in quality of life (Wang et al., 2024). For these reasons, ET is the most widely recommended treatment for obesity in humans (Oppert et al., 2023). The current study focuses on the molecular mechanisms underlying the beneficial effects of 6 weeks of ET in a female rat genetic model of obesity, in which a mutation in the gene encoding the leptin receptor leads to increased appetite (Paracchini et al., 2005). We revealed distinct patterns in the activation of two neurotrophin-stimulated intracellular signalling pathways (PLC/PKC/CAMKII and PI3K/Akt) in the hippocampi of obese, trained lean, and trained obese animals. These alterations are associated with differential expression of both pre- and post-synaptic markers (SYP and LRRC4) crucial for synaptic plasticity, as well as markers identifying various cell types. Six weeks of ET increases the expression of CAMKII and SYP in obese rats, upregulates regeneration-related transcripts (CNP, PLP1, and NF-1) and improves cognitive functions. Moreover, in this experimental group, we found a high- to a very high positive correlations (r = 0.7–0.9 and r = 0.9–1.0 respectively) between BDNF and components of the RAF/ERK1/2 signaling pathway, between RAF/ERK1/2 and astrocyte marker, as well as two regeneration parameters (NF-1 and GAP-43). These results provide evidence that six weeks of moderate exercise benefits obese rats by regulating the gene expression of growth factors and signaling pathways essential for brain plasticity.

### 4.1 Activation of three major signalling pathways after 6 weeks of ET in the hippocampus of lean and obese animals

The neurobiological effect of BDNF after binding to the TrkB receptor depends on which signalling pathway is modulated. To date, several neurotrophin-dependent signalling pathways—PI3K/Akt, PLCγ/PKC/CAMKII, and RAF/ERK1/2—have been identified in neural tissue, each responsible for distinct outcomes (Sasi et al., 2017).

The PI3K/Akt pathway plays a crucial role in maintaining metabolic homeostasis, promoting cell proliferation and survival, and mediating anti-apoptotic effects (Lou et al., 2011, Sánchez-Alegría et al., 2018).

Here, we show that 6 weeks of ET had a significant impact on PI3K/Akt transcripts in the hippocampus of lean rats. This was associated with increased expression of microglial and astrocyte markers, pre- and post-synaptic markers (SYP and LRRC4), and an axonal growth marker (GAP-43), suggesting that ET modulates hippocampal synaptic transmission. Recently, Gao et al. (2023) reported increased mRNA levels of PI3K, Akt, and mTOR following a specific treadmill training programme in rats. This upregulation contributed to hippocampal neuronal cell protection and enhanced autophagy, exerting an anti-apoptotic effect. Similarly, Peng et al. (2022) confirmed that an 8-week ET regimen exerts neuroprotective effects via the PI3K/Akt pathway by suppressing the expression of GSK3β—a protein involved in neural apoptosis—while simultaneously improving hippocampal morphology and memory performance. Our findings further revealed that obese controls expressed the Akt gene to a greater extent than its upstream activator, PI3K. We propose that alternative Akt-activating kinases, which respond to growth factors, inflammatory cytokines, or DNA damage (Mahajan and Mahajan, 2012), may stimulate Akt independently of PI3K. Such activation supports the pro-survival role of Akt by inactivating pro-apoptotic proteins (Van Der Heide et al., 2006) and upregulating anti-apoptotic proteins (Pugazhenthi et al., 2000). Given that the positive effects of ET on obesity-induced oxidative stress have already been reported (Groussard et al., 2019), we hypothesise that the decrease in PI3K/Akt gene expression observed six weeks after ET in obese rats may reflect the suppression of obesity-related negative effects. Moreover, healthy cells have been shown to prevent prolonged Akt activity through proteasomal degradation (Adachi et al., 2003).

PLCγ is the key upstream component that responds to the activation of neurotrophin receptors by transmitting signals to two independent downstream targets: PKC and CAMKII (Clapham, 2007, Huang and Reichardt, 2003). These downstream effectors are well known for their prominent role in synaptic plasticity. Of the two, CAMKII plays a central role in controlling this plasticity and long-term potentiation (LTP), an activity-dependent strengthening of synapses that is crucial for learning and memory formation (Xiao et al., 2023, Yasuda et al., 2022). Our findings indicate that ET is most effective in increasing PLCγ/CAMKII gene expression in lean rats, followed by obese counterparts, suggesting its potential to support neuroplasticity. Vaynman et al. (2004) identified a link between the TrkB receptor and CAMKII activity; when rats were subjected to short-term physical activity and the TrkB receptor was blocked, the effect of exercise on CAMKII, synapsin-I, mitogen-activated protein kinase II, and spatial memory was abolished. Their follow-up study (Vaynman et al., 2007) also showed that blocking CAMKII prevented exercise-induced expression of BDNF and its downstream transcription factor, CREB, suggesting that CAMKII plays a sophisticated role in modulating the cognitive effects of training. The negative impact of obesity on CAMKII gene expression observed in the present study may indicate disruption of LTP, synaptogenesis, and plasticity. Using the same genetic model of obesity, Alzoubi et al. (2005) reported reduced total and phosphorylated protein levels of CAMKII in the hippocampal CA1 region. Our results show that moderate ET positively affects the restoration of CAMKII gene expression, potentially strengthening synaptic plasticity. Furthermore, our findings reveal that obesity alone increases mRNA levels of both PLCγ and its other downstream target, PKC. This genetic model of obesity is characterised by hyperinsulinaemia and insulin resistance (Durham and Truett, 2006), which results in altered hippocampal synaptic plasticity and impaired spatial learning (Grillo et al., 2015). This impairment is mediated by overactivation of PKC (Tangvarasittichai, 2015) leading to sustained stimulation of mitogen-activated protein kinases, including ERK1/2 (Jubaidi et al., 2022). Such a signalling cascade disrupts the interaction between the insulin receptor substrate and the insulin receptor itself, thereby negatively affecting synaptic plasticity (Copps and White, 2012). Oxidative stress is also recognised as a direct consequence of overnutrition-induced obesity (Qiu and Schlegel, 2018) because the increased supply of glucose and uptake of free fatty acids by the brain place a strain on the mitochondrial respiratory chain (Rebelos et al., 2020). Elevated diacylglycerol synthesis from incoming free fatty acids can activate PKC, which in turn activates NADPH oxidase, inducing reactive oxygen species production (Inoguchi et al., 2000). This process can increase gene expression of PKCδ and promote neuronal cell death via apoptosis (Jin et al., 2011). Previously, Emami et al. (2016) reported the efficacy of ET as an intervention against obesity-induced systemic oxidative stress, and Koo and Kang (2019) further confirmed its role in improving redox status in hippocampal tissues. It appears that moderate six-week ET may counteract obesity-induced oxidative stress, at least in part, through the reduction of PKC mRNA.

Our results further show that the obese control group exhibits elevated mRNA levels of RAF/ERK1/2 signalling. In addition to insulin resistance, several other negative factors may contribute to the overexpression of ERK1/2 in obesity, including high blood glucose, oxidative stress, inflammation, and apoptosis—each closely linked to hippocampal atrophy in obese individuals (Kassouf and Sumara, 2020, Lee and Yau, 2020, Yu et al., 2011). Both lean and obese rats in our study showed a notable increase in the expression of all selected components of this signalling pathway following the training programme. At first glance, this may appear counterproductive based on the aforementioned factors; however, under certain conditions, ERK1/2 signalling can exert both pro- and anti-apoptotic effects (Yue and López, 2020). Consistent with our findings regarding ERK1/2 expression after ET, Cai et al. (2016) reported that obesity-induced apoptosis was reversed following aerobic exercise intervention, which was associated with a marked increase in ERK activity and mediated an anti-apoptotic response. It is well established that ET is not limited to effects within the hippocampus, but rather induces systemic changes. Skeletal muscle, in particular, is strongly affected by ET and produces muscle-derived hormones known as myokines. Li et al. (2017) proposed that the novel exercise-induced hormone irisin is involved in activating both ERK and Akt signalling, thereby contributing to neuroprotection. Although statistical significant difference was not observed between the obese groups in our study, we hypothesize that ET contributes to the maintenance of ERK1/2 mRNA levels in trained obese animals. Correlation analysis underscores the importance of RAF/ERK1/2 signaling in astrocytes, which play a critical role in maintaining central nervous system homeostasis. Moreover, this signaling pathway is associated with changes in regeneration markers NF-1 and GAP-43, which contribute to neuronal structural integrity and adaptive processes mediated by RAF/ERK1/2 signaling. Nevertheless, the possibility of a synergistic interaction between obesity and ET in triggering RAF/ERK1/2 gene expression warrants further investigation.

### 4.2 Increased expression of pre- and post-synaptic markers in the hippocampus of trained rats

Six weeks of ET had a significant impact on the upregulation of both pre- and post-synaptic markers (SYP and LRRC4), with a more pronounced effect on SYP. SYP is located in the membrane of synaptic vesicles and facilitates their fusion with the pre-synaptic plasma membrane, leading to the release of neurotransmitters into the synaptic cleft (Valtorta et al., 2004). CAMKII, a downstream target of PLCγ, contributes to this process by phosphorylating several vesicle-associated proteins, including SYP (Rubenstein et al., 1993). Our training programme resulted in the upregulation of PLCγ and CAMKII mRNA, while promoting the dominant expression of SYP over LRRC4. This finding may indicate a reciprocal relationship between CAMKII signalling and SYP in the processes of LTP, synaptogenesis, and plasticity. Supporting this, Cai et al. (2016) confirmed that ET restored hippocampal plasticity-related protein levels of SYP following obesity-induced downregulation of genes and proteins associated with neuronal plasticity.

Given that cognitive-related brain areas such as the hippocampus have a wide distribution of insulin receptors, impaired insulin signalling may contribute to the disruption of hippocampal neuroplasticity (Fadel and Reagan, 2016). Li et al. (2019) found that diabetic mice with altered insulin signalling exhibited decreased levels of SYP protein, which were ameliorated by an aerobic exercise intervention. We confirm a beneficial role of 6 weeks of ET in restoring components crucial for hippocampal neuroplasticity. Both trained groups showed the same effect of ET in promoting synaptic plasticity, as indicated by hippocampal expression of CAMKII and SYP genes. Considering the observed changes related to synaptic plasticity and LTP, we conducted a passive avoidance test to assess learning and memory modulation. Although latencies were generally shorter than expected, trained obese animals showed notable improvements. However, interpreting these results requires careful consideration of the cognitive processes involved. The level of cognitive flexibility may significantly affect performance in the passive avoidance test. Chaby et al. (2019) found that animals undergoing cognitive flexibility training exhibited accelerated extinction learning during retention testing, suggesting an enhanced capacity to adapt to changing contexts. Short latencies often imply difficulties in adapting behaviour following negative experiences, potentially indicating impairments in learning, memory, or executive function. Interpreting these outcomes necessitates a holistic approach, taking into account psychological, neurological, and environmental factors. Our findings suggest that variability in latencies—and the overall short latencies observed—may be influenced by factors related to cognitive flexibility, extinction processes, or differences in synaptic marker transcript levels. The ET protocol used in this study confirmed molecular changes associated with learning and memory. In support of this, Inoue et al. (2014) demonstrated that 6 weeks of mild exercise significantly enhanced memory performance in rats, as assessed by the Morris water maze. We therefore propose that the variability in our samples and the generally short latencies are not indicative of an insufficient training programme. It is widely recognised that incorporating physical activity into daily life improves cognitive function, particularly memory and learning. Notably, the molecular changes associated with exercise can manifest relatively quickly. Understanding how these changes interact with cognitive flexibility and extinction processes will be important for future studies.

The post-synaptic marker LRRC4 is involved in the formation of excitatory synapses and axonal differentiation (Deng and Wu, 2023). Unfortunately, there is a lack of evidence in the literature on how ET influences changes in LRRC4 expression in the hippocampus. One of the few indications that ET promotes LRRC4 expression comes from treadmill training after spinal cord hemisection in rats (Kobayakawa et al., 2019), where the authors observed upregulation of 36 synapse-related genes, with LRRC4 being the most prominently expressed. We demonstrate, for the first time, that ET in lean rats increases LRRC4 mRNA levels in the hippocampus. Xu et al. (2015) have already shown that LRRC4 regulates the local stabilisation of microtubules and promotes axonal outgrowth via the aPKCζ/microtubule affinity-regulating kinase 2 pathway. Rahmati and Kazemi (2019) provided evidence that mild-intensity treadmill exercise is optimal for significantly increasing GAP43—a gene related to growth and plasticity in the hippocampus—compared with low- or high-intensity training. Similarly, a study by Cheon and Koo (2013) confirmed the positive effect of forced or voluntary exercise on GAP43 expression in the hippocampus of aged rats. However, in our study, LRRC4 gene expression was not positively affected in obese and/or trained obese rats. When discussing changes in the hippocampus of obese animals, we must consider systemic alterations, including low-grade inflammation, which impairs synaptic plasticity and cognitive function, along with disrupted lipid and glucose metabolism (Erion et al., 2014, Salas-Venegas et al., 2022, Singla, 2010). Although LRRC4 appears to be critical for the development of pathway-specific synapses and the functional integration of distinct inputs in the hippocampus (DeNardo et al., 2012), many questions remain unresolved in this context.

### 4.3 ET in obese rats activates processes involved in tissue repair and regeneration

Oligodendrocytes, particularly mature ones, play a fundamental role in the formation of the myelin sheath around axons. Their involvement in myelin production and axonal support is well documented (Kipp, 2020, Mathis et al., 2001). A recent study suggested that exercise, rather than pharmacological intervention, may be responsible for promoting oligodendrocyte differentiation and enhanced myelination (Tang et al., 2021). In contrast to lean rats, obese counterparts in our study exhibited increased gene expression of both oligodendrocyte markers (CNP and PLP1) following ET. This effect may be linked to anti-inflammatory immunomodulation, as evidenced by the observed decrease in activated microglia (IBA1) and/or an upregulation of growth factors induced by exercise. Oligodendrocytes are particularly sensitive to oxidative stress because of their exceptionally high energetic demands for sustaining myelination processes (Rosko et al., 2019). Combined with a high-fat diet, oxidative stress-induced loss of oligodendrocytes in the central nervous system has been reported (Langley et al., 2020). ET may therefore serve as an effective intervention in obese animals to prevent oligodendrocyte loss by promoting mitochondrial homeostasis (Graham et al., 2019, Yoon et al., 2016).

## 5 Conclusions

Overall, ET of moderate intensity has been shown to be an effective endogenous intervention that counteracts the negative effects of obesity in the hippocampus. In this study, we demonstrate the potential of ET to modulate neurotrophin-induced mRNA expression of signalling pathway components involved in synaptic plasticity, LTP, neuroprotection, and metabolic homeostasis. Following ET, obese animals exhibited increased expression of CAMKII and the presynaptic marker SYP, decreased levels of the activated microglia transcript Iba1, and positive changes in markers of both mature and immature oligodendrocytes and neurofilaments—indicating their involvement in supporting regenerative processes. The activation of these mechanisms was accompanied by partial improvement in cognitive function, as assessed by the passive avoidance test.

## CRediT authorship contribution statement

**Karolína Kuchárová:** Writing – review & editing, Validation, Methodology, Funding acquisition. **Lenka Ihnátová:** Validation, Software, Methodology. **Katarína Kiss Bimbová:** Methodology, Formal analysis. **Alexandra Kisucká:** Software, Methodology, Funding acquisition, Data curation. **Mária Ileninová:** Software, Methodology, Data curation. **Nadežda Lukáčová:** Writing – review & editing, Writing – original draft, Supervision, Funding acquisition, Formal analysis, Conceptualization. **Martina Magurová:** Software, Methodology. **Ján Gálik:** Writing – review & editing, Conceptualization. **Tomáš Kuruc:** Writing – review & editing, Writing – original draft, Visualization, Validation, Software, Methodology, Investigation, Formal analysis, Data curation, Conceptualization.

## Compliance with ethical standards

The experimental procedures were approved by the State Veterinary and Food Administration of the Slovak Republic (decision No. 4434/16–221/3), as well as by the Animal Use Committee at the Institute of Neurobiology, Biomedical Research Centre of the Slovak Academy of Sciences, in compliance with the EC Council Directive (2010/63/EU) on the use of animals in scientific research. The study adheres to the 2020 AVMA and ARRIVE euthanasia standards.

## Funding

This research was supported by the Research and Development Operational Programme funded by the 10.13039/501100008530European Regional Development Fund (IMTS: 313011V344), the Slovak Research and Development Agency (APVV-19–0324), and the Grant Agency of the Ministry of Education, Science, Research and Sport of the Slovak Republic and the Slovak Academy of Sciences (VEGA 2/0115/24 and VEGA 2/0117/24).

## Declaration of Competing Interest

The authors declare that they have no known competing financial interests or personal relationships that could have appeared to influence the work reported in this paper.

## Data Availability

The datasets used or analyzed during the current study are available from the corresponding author on reasonable request.
